# From forest to fragment: compositional differences inside coastal forest moth assemblages and their environmental correlates

**DOI:** 10.1007/s00442-021-04861-7

**Published:** 2021-02-01

**Authors:** Britta Uhl, Mirko Wölfling, Konrad Fiedler

**Affiliations:** Department of Botany and Biodiversity Research, University of Vienna, Rennweg 14, 1030 Vienna, Austria

**Keywords:** Mediterranean insects, β-Diversity, Species turnover, Land use, Conservation areas

## Abstract

Patterns of β-diversity can provide insight into forces shaping community assembly. We analyzed species-rich insect assemblages in two reserve fragments that had once been part of one contiguous Mediterranean coastal pine forest. Local environments are still similar across both fragments, but their landscape context differs strongly, with one surrounded by intense agricultural land, while the other neighbors the urbanized area of Ravenna. Using 23,870 light-trap records of 392 moth species, and multiple local and landscape metrics, we compared the relative importance of habitat- versus landscape-scale environmental factors for shaping small-scale variation in differentiation and proportional insect β-diversity across 30 sites per reserve. Moth assemblage composition differed substantially between fragments, most likely due to ecological drift and landscape-scale variation. For proportional β-diversity, especially local forest structure was important. At well-developed forest sites, additive homogenization could be observed, whereas the lack of typical forest species at dry, dense, and younger forest sites increased species turnover (subtractive heterogenization). For differentiation β-diversity, local and landscape-scale factors were equally important in both reserves. At the landscape-scale (500 m radius around light-trapping sites) the proximity to urban areas and the fraction of human-altered land were most important. At the habitat scale, gradients in soil humidity, nutrient levels and forest structure mattered most, whereas plant diversity had very little explanatory power. Overall, landscape-scale anthropogenic alterations had major effects on moth communities inside the two conservation areas. Yet, even for these parts of one formerly contiguous forest trajectories in community change were remarkably idiosyncratic.

**Supplementary Information:**

The online version contains supplementary material available at 10.1007/s00442-021-04861-7.

## Introduction

Recently, various studies have reported drastic insect declines across landscape levels (Habel et al. 2019a; van Klink et al. 2020). Human actions like land-use change and intensification are major drivers of species losses in urban and agricultural landscapes (Allan et al. 2015; Newbold et al. 2016). Yet, Seibold et al. (2019) and Hallmann et al. (2017) found that severe insect decline is also detectable inside conservation areas. There, local as well as landscape-scale correlates have been shown to be associated with local variation in the diversity of insect communities (Uhl 2020). Inside nature reserves, anthropogenic actions might indirectly influence population dynamics through fragmentation and isolation effects (Habel and Schmitt 2018; Rossetti et al. 2017) or alter communities by nutrient and pollutant drift (Botías et al. 2016; van Dobben and de Vries 2017). In fact, most conservation areas nowadays exist as isolated fragments, surrounded by human-modified areas. With fragmentation, gene flow between habitat patches can become interrupted (Habel and Schmitt 2018). Some species might not persist in the long run within isolated patches, depending on their life-history traits (Slade et al. 2013). As a result of this directional environmental filtering combined with stochastic ecological drift, anciently connected communities might diverge over time, forming new assemblages with species adapted to survive under the circumstances of the according habitat patch (Vellend 2016).

While the recent insect decline debate is often focusing on species richness and biomass, there is a lack in studies investigating community composition and species turnover. Species composition of ecosystems can give important insights into environmental change (Dornelas et al. 2014; Mendenhall et al. 2012). In fact, impacts on ecosystems primarily are reflected by changes in community composition, as species sharing certain traits might be filtered out and replaced by others (Dornelas et al. 2014; Slade et al. 2013). Losses in α-diversity, in contrast, might only occur with delay, when significant changes in species assemblages might already have impacted ecosystem function (Mori et al. 2018). Changes of the local habitat structure and differing management regimes can affect species composition (for butterflies and moths: Fies et al. 2016; Mangels et al. 2017; Truxa and Fiedler 2012). Furthermore, communities can be altered by landscape-scale changes such as landscape simplification (Gámez-Virués et al. 2015) or increasing anthropogenic influence due to pollution or eutrophication (Uhl et al. 2016; WallisDeVries and van Swaay 2017).

Quantifying the compositional change in communities needs a clear definition, as it is often mixed up with other aspects of β-diversity. In fact, there are various interpretations of β-diversity, leading to multiple β-diversity indices, which address different aspects of compositional variation (Tuomisto 2010a, b). Following its original definition, β-diversity describes a multiplicative or additive partitioning value, by putting α-diversity in context to larger-scale γ-diversity (Anderson et al. 2011). Jurasinski et al. (2009) suggested summing up such measures as “proportional diversity” measures. Tuomisto (2010a) in contrast suggested calling the multiplicative partitioning of β-diversity “true beta diversity”, as it is most likely fitting the classical definition, while additive β-diversity should be called “regional diversity excess”. Proportional β-diversity, or true β-diversity, is a correlate to α-diversity, putting the local species diversity in relation to the regional γ-diversity. However, more commonly β-diversity is used in the sense of differentiation diversity i.e. variation in species composition between sites (Anderson et al. 2011; Jurasinski et al. 2009). By partitioning the variation in community structure as a response to environmental factors, differentiation diversity can give insight into how much of observed community change in space or time can be explained by environmental variation (Anderson et al. 2011). We here analyze both, proportional β-diversity and differentiation diversity, in an attempt to unravel the influence of a variety of environmental factors on these two complementary aspects of β-diversity.

First, we want to investigate, how insect assemblages of two anciently connected Mediterranean forest nature reserves nowadays differ in their composition and proportional β-diversity. By analyzing multiple environmental variables, we also try to unravel which ecological filters likely caused this divergence. Second, we are interested in the relative importance of different sets of environmental characters, shaping variation in community composition within each of the two reserves. Especially the potential influence of human actions outside the conservation areas, such as agricultural land use and the proximity to urbanized areas, is considered. Our main research hypotheses therefore are:

- The moth assemblages of the two reserves today differ significantly from another, although both reserves share the same history and provide similar habitats.
- Proportional β-diversity informs about the environmental drivers shaping community assembly on the small scale. A well-developed forest structure should provide more niches and, therefore, favor the occurrence of larger subsets of the regional species pool (additive homogenization). Potential pollution sources otherwise might cause subtractive heterogenization, as species get lost from the local assemblages.
- Looking at differentiation diversity, effects of both—local and landscape-scale factors—are reflected by the small-scaled moth community composition. However, to understand how these factors shape moth communities, one has to look at the occurrence patterns of individual species and their traits.

As a target group, we selected nocturnal Lepidoptera (‘moths’) since these terrestrial insects are usually rich in species, can easily be sampled using light traps, and reflect a wide variety of bionomic strategies (Slade et al. 2013; Summerville and Marquis 2017). At the same time, moths show close functional links to the vegetation of their habitats, mostly through the nutritional demands of their larval stages. Accordingly, a plethora of studies revealed that species composition of moth assemblages usually closely tracks environmental variation down to small spatial scales (Guariento et al. 2020; Habel et al. 2019b; Wölfling et al. 2019).

## Methods

### Study sites

Our study sites were located within two Mediterranean coastal forest reserves in North-Eastern Italy, near Ravenna. The reserves Pineta san Vitale (hereafter PsV) and Pineta di Classe (herafter PdC) once were part of one big coastal forest area, covering an area of approximately 6000 ha (Malfitano 2002). However, after 1796, deforestation due to land-use change and the development of the city and harbor of Ravenna lead to the disappearance of most of the former natural forest area. Nowadays, only about 2000 ha, split up between the two disconnected reserves, remain (Andreatta 2010; Malfitano 2002). As a part of the regional park Po Delta, they both are listed as UNESCO biosphere reserves and are also partly considered as important bird areas, wetlands of international importance following the convention of Ramsar, and Natura 2000 sites.

The more northern reserve PsV has a total area of about 950 ha and directly neighbors the industrial harbor of Ravenna. To the east, the lagoon Pialassa Baiona forms the border of the reserve, whereas other near-natural wetland areas adjoin to the north and north-west of PsV. In the south-west, agricultural fields and other anthropogenically modified areas neighbor the reserve. The vegetation of PsV mostly consists of mixed oak and pine woods, but also reed areas, open grassland, and riparian forest. Therefore, PsV is a structurally rich near-natural reserve with many different vegetation types, offering typical Mediterranean warm and dry habitats on the one hand, but also riparian and wetland areas with more humid conditions on the other (Merloni and Piccoli 1999).

PdC, the more southern forest reserve, is about 10 km away from PsV, has a total area of about 900 ha, and is mostly surrounded by agricultural areas. Only in the south-east of PdC, near-natural wetland areas adjoin the reserve. As in PsV, the main vegetation type of PdC is mixed oak and pine woods. However, this reserve has not as much structural heterogeneity as PsV and local conditions seem to be drier, as indicated by the vegetation (Uhl et al. 2020a). Additionally, some pine forest parts in the center of PdC are quite monotonous, with impoverished plant diversity and no other habitat structures in their surroundings (Piccoli and Merloni 1999). In the south-west, very dense and young pine forest stands can be found, indicating more recent reforestation activities from about 30 years ago (Enrica Burioli, pers. communication).

Within each of the two reserves, 30 sampling sites (60 sites in total) with on average 821 m distance to each other (SD ± 280 m) were chosen. By doing so, we wanted to achieve equal distribution of sampling points throughout the reserves. All locations were situated in mixed oak and pine forest to ensure comparability of the habitats where the samples had been taken. Furthermore, sites were selected in such a way that in a radius of about 100 m no other vegetation types occurred prominently. Locations had to be accessible by car and were always placed at small forest gaps so that no bushes and trees could hinder light emission of the light traps used for moth sampling.

### Data sampling

#### Landscape-scale data

We analyzed landscape structure at two different ranges (200 m and 500 m radius) around each light trap site, based on aerial photographs taken in the year 2017, as provided by Google Maps™. This was done to see which spatial scale effect of the surrounding landscape was most influential in moth communities. The 200 m range represents the small-scale surroundings, while the 500 m range stands for the large-scale context extending into the landscape outside of the reserves. Within each perimeter, we quantified landscape elements using the program QGIS (QGIS Development Team 2018). In particular, the proportions of forest, reed and open grassland areas were measured, as well as the proportion of areas covered by open water bodies, agricultural fields, and urban/industrial areas. The latter two ones were summed up as “human-modified areas” in subsequent analyses. Based on the area fractions of forest, reed and grassland areas, the diversity of natural habitat areas was calculated, using the Shannon index. Edge density (in m/ha) served as a measure for landscape fragmentation. Additionally, the distance of moth sampling sites to the nearest forest edge, industrial area and water canal was measured.

#### Vegetation sampling

Vegetation was sampled within five 1 × 1m^2^ plots for herb layer, and five 5 × 5m^2^ plots for shrub layer at each site. In each of these herb and shrub layer plots, every plant species was identified and listed in an incidence matrix. Forest structure was analyzed by doing ten point-centered-quarter (PCQ) analyses per sampling site, following Mitchell (2010). Each tree that was included in the PCQ-analysis was identified to species level. Out of the PCQ-data, we were able to calculate forest density (in trees ha^−1^), cover of deciduous trees (in m^2^ ha^−1^), cover of conifer trees (in m^2^  ha^−1^), mean basal area of trees (in m^2^), and the standard deviation of basal areas. Additionally, canopy density was recorded by using a forest densiometer (Forest densiometers, Robert E. Lemmon, Rapid City). The proportion of dead standing trees was estimated by sight. From the aggregated plant species incidence data (herbs, shrubs, and trees), we calculated plant species richness per plot. As a measure of β-diversity among the vegetation, multivariate dispersion for the herb and shrub layer was calculated for each site (Anderson et al. 2006). Functional dispersion of plants was also calculated, following Laliberté et al. (2014), using the plant incidence data and a matrix with collated trait information as described in Uhl et al. (2020a). Furthermore, plant indicator values after Ellenberg were collected from Pignatti et al. (2005). From these latter data, we calculated a mean indicator value for soil nutrients, humidity and temperature for every light-trap site. Further information on vegetation sampling can be found in Uhl et al. (2020a).

#### Moth sampling

Moths were sampled using automated light traps as described in Axmacher and Fiedler (2004). We used two 18 W light tubes (one Sylvania black light and one white black light tube) as light source, powered by 12 V dry battery packs. Start of the sampling was at dusk with a sampling duration of 6–8 h per night. Data collection took place from 2015 to 2017 in May and June for the early summer moth communities and in August for the late summer moth communities. Each year, we sampled 20 randomly chosen sites out of the 60 locations, avoiding full moon periods and spells of rain, as both these factors may strongly affect flight behavior of moths (Yela and Holyoak 1997). Subsequently, all moths captured in the traps were identified to species level, aggregated per site, and the resulting abundance-weighted species × site matrix served as the basis for all explorations of moth diversity (see Uhl et al. 2020b for further details).

### Data analysis

As a first step, we analyzed the differences in moth composition between the two anciently connected reserves. This was done by identifying indicator species for each reserve via the ‘indval’ function, as included in the R package ‘labdsv’ (Roberts 2016). We compared environmental variables of PsV and PdC to determine candidate predictors potentially responsible for the divergence of the two forest moth assemblages using Mann–Whitney *U* tests, adjusted for multiple comparisons by false discovery rate control (Benjamini and Hochberg 1995; Pike 2011). These tests were only intended to illustrate differences in small-scaled environmental factor variability between the two reserve fragments and should not be interpreted as valid hypothesis tests (as with the classical interpretation of *p* values).

For the further analyses of small-scale variation in community composition (differentiation diversity) and proportional β-diversity, we did not use the raw environmental factors but rather condensed these into principal component axes (PC-axes). This was done to avoid collinearity and to reduce a large number of potential environmental predictors. Principal Component Analyses (hereafter PCA) were performed separately for the local and landscape-scale variables. Assuming that different environmental conditions might be differentially important for the two reserves, we calculated reserve specific local and landscape PCAs for PsV and PdC separately. So in total, four PCAs (local-PsV, local-PdC, landscape-PsV, landscape-PdC) with varimax rotation were performed in the R environment using the package ‘psych’ (Revelle 2018). In the local PCA, 14 factors were included as variables (Online Resource 2). In the landscape-scale PCA, 8 factors were included (Online Resource 3). The number of the extracted PC-axes was determined through the Kaiser criterion. The resulting PC-axis scores of sampling sites then served as predictors in linear models and in multivariate ordinations of the local moth communities (see below).

Using the moth community data, we calculated the exponential Shannon α-diversity for each sampling site. Additionally, γ-diversity was calculated the same way, but with moth data from all 30 locations per reserve pooled. By doing so, we received two γ-diversity values, referring to either PsV or PdC. As we were especially interested in partitioning diversity into proportional fractions, we decided to use the proportional species turnover (viz. *β* = 1−*α*/*γ*) as a measure for proportional β-diversity (Tuomisto 2010a). This β-diversity index is a multiplicative partitioning method defining local assemblages as fractions of the regional species pool. By dividing the observed local species diversity fraction from 1, the index becomes a measure for “turnover”, matching the original definition of β-diversity. So, small values of this β-diversity imply that the local community is near as species rich as the entire region (based on large species subsets), while larger values indicate that locally, only minor fractions of the all-over γ-diversity can be found. Small β-values, therefore, indicate small species turnover, while larger values indicate a rather heterogeneous representation of species across sites.

As the local proportional β-diversity values (*β*_observed_) are all dependent on the regional γ-diversity, there is interdependence between the observed β-diversity values. To correct for the effect of this dependency, we additionally calculated the standardized β_dev_ as suggested by Mori et al. (2015). Using a null model with fixed species occurrence frequencies and randomizing 999 times, we calculated the mean null distribution of β-diversity (*β*_null_) and the SD of the null distribution (β_SD_). The standardized β-diversity β_dev_ is defined as (*β*_observed_—*β*_null_)/*β*_SD_ and can inform about “the magnitude of deviation from the expected β-diversity in a random assembly process” (Mori et al. 2015).

Standardized β-diversity (*β*_dev_) served as response variable in linear models, where the PC-axes of the environmental variables were used as predictors. Models were calculated in the R workspace using the ‘nlme’ package (Pinheiro et al. 2018). Best model selection was done via the Akaike information criterion and the ‘stepAIC’ function of the ‘MASS’ package (Venables and Ripley 2002). Additionally, we tested for significant differences between the PsV and PdC β-diversity values. Like for the environmental variables, we, therefore, used the Mann–Whitney *U* test.

Looking at differentiation β-diversity, we tested if there is a significant difference between the reserve specific moth communities. For this, a Bray–Curtis similarity matrix was calculated using the square-root transformed abundance data of all 60 sites. The used permutation test was calculated via the ‘adonis’ function from the package ‘vegan’ in R (Oksanen et al. 2018). To analyze the potential effect of environmental factors on local moth community composition, we performed a Canonical Analysis of Principal coordinates (CAP) using the ‘vegan’ package in R (Oksanen et al. 2018). The two reserves here were treated separately. The site scores along the first three PC-axes of the local PCA, served as explanatory variables. From the landscape PCA, site scores of the ‘Habitat diversity’-, the ‘modified areas’-axis and the ‘Distance to industry’-axis were used as predictors. All predictors were z-transformed for standardization. For assessing the significance of correlations, we used a PERMANOVA test with 999 randomizations.

## Results

In total, we found 23,870 individuals of 392 moth species. 259 of these species (66.1%) were found in both reserve fragments, while 81 species (38 of which were singletons) only occurred in PsV, and 52 species (22 singletons) were exclusive to PdC. So, for PsV we found 340 species, while in PdC only 311 species were recorded. The exponential bias-corrected Shannon α-diversity for all sites was on average higher in PsV (43.6 ± 10.7) than in PdC (38.2 ± 9.9). γ-diversity of both reserve fragments, expressed by the same metric, reached roughly equal values (PsV: 75.2, PdC: 77.9).

Typical moth species of PsV, extracted via the indval-function, included specialist oak feeders like *Teleiodes luculella* and *Acrobasis consociella*, but also the highly polyphagous *Clepsis consimilana* and *Ligdia adustata* (host-specific to *Euonymus* shrubs) emerged as indicators. For PdC, the moss-feeding *Eudonia mercurella*, the pine herbivore *Macaria liturata*, and the oak feeder *Spatalia argentina* were characteristic. All indicator species, having a probability of > 0.05 to preferentially appear in only one reserve fragment, are listed in Online Resource 1.

Comparing the small-scaled variation in environmental factors, only local plant diversity per site differed substantially between the two reserves, being on average higher at sites in PsV. Trees also were on average larger and the forest was more heterogeneous there. In contrast, we found marginally more trees/ha and on average more dead wood in PdC. At the landscape level, sampling sites in PsV had higher habitat and landscape diversity and contained more reed areas. Furthermore, in PsV there are more water canals, as shown by smaller distances from each sampling site to the closest canal (Table 1).

**Table 1:** Mean values and standard deviation of the environmental variables measured at 60 light-trapping sites situated in the two forest reserve fragments PsV and PdC in north-eastern Italy

	Reserve PsV	Reserve PdC	*t*/*z* value	*p* value
**Local site characteristics**				
Plant species richness	36.6 ± 5.7	33.0 ± 5.3	– 2.47	**0.01**
Functional dispersion of plant species	0.18 ± 0.01	0.18 ± 0.01	– 1.38	0.17
Herb layer heterogeneity	0.38 ± 0.1	0.37 ± 0.1	– 0.87	0.38
Shrub layer heterogeneity	0.25 ± 0.1	0.25 ± 0.1	– 0.73	0.47
Ellenberg indicator “Humidity”	4.2 ± 0.1	4.1 ± 0.1	– 1.39	0.16
Ellenberg indicator “Nutrients”	4.4 ± 0.2	4.2 ± 0.2	– 1.86	0.06
Ellenberg indicator “Temperature”	6.5 ± 0.1	6.5 ± 0.07	– 0.94	0.35
Forest density (mean trees/ha)	308.3 ± 121.1	345.5 ± 103.0	– 1.53	0.13
Canopy density (in %)	64.3 ± 13.8	65.4 ± 14.8	– 0.34	0.73
Cover of deciduous trees (m^2^ ha^−1^)	11.8 ± 7.1	11.3 ± 5.4	– 0.29	0.77
Cover of conifer trees (m^2^ ha^−1^)	12.9 ± 7.4	13.8 ± 8.2	– 0.29	0.77
Mean basal area (in cm^2^ ha^−1^)	897.5 ± 357.4	755.4 ± 238.4	– 1.42	0.16
Standard deviation of basal area	1107.4 ± 411.7	946.3 ± 327.9	– 1.78	0.08
% dead standing trees	8.4 ± 8.2	11.4 ± 11.3	– 1.09	0.28
**Landscape-level characteristics**				
Distance to reserve edge (in m)	424.6 ± 264.9	419.5 ± 275.0	– 0.14	0.89
Distance to canal (in m)	188.5 ± 218.2	593.3 ± 476.7	– 4.07	** < 0.001**
Distance to industry (in m)	4035.1 ± 1186.9	13,583.0 ± 1556.5	– 6.65	** < 0.001**
Diversity of natural habitats (200 m)	0.40 ± 0.19	0.15 ± 0.20	– 4.48	** < 0.001**
Edge density (500 m)	62.3 ± 21.0	43.4 ± 33.3	– 2.61	**0.01**
Proportion of reed (200 m)	0.09 ± 0.10	0.02 ± 0.10	– 3.70	** < 0.001**
Proportion of grassland (200 m)	0.04 ± 0.10	0.01 ± 0.02	– 2.49	**0.01**
Proportion of modified areas (500 m)	0.08 ± 0.12	0.13 ± 0.14	– 1.61	0.11

The *t*/*z* values and the *p* values of Mann–Whitney *U* tests are also given

Results printed in bold face were statistically significant (at *p* < 0.05) after table-wise False Discovery Rate correction

### Multivariate description of site characters

The PsV-local-PCA resulted in five PC-axes with eigenvalues > 1.00, together explaining 76% of the variation. Axes were named after their main factor loadings to facilitate interpretation (Online Resource 2). The PdC-local-PCA also resulted in five PC-axes to be selected, explaining 77% of the variation. In contrast to the PsV-PCA, the factor loadings of the five PC-axes were sorted differently, leading us to attribute alternative axis names to them (Table 2). In the landscape-PCA, the four first axes explained 84% of the variation in PsV. In PdC, only two axes were extracted, following the Kaiser criterion. However, these two axes explained 70% of variation (Table 2, Online Resource 3 for factor loadings).

**Table 2:** Names of the ordination axes with Eigenvalues > 1.00 which resulted from the different PCAs in order to condense raw variables

	PC-Axis 1	PC-Axis 2	PC-Axis 3		PC-Axis 4		PC-Axis 5		Total expl. variation
PCA of local factors									
PsV	Old, open forest (20%)	Plant diversity (18%)	Humidity nutrient gradient (17%)		Herb-layer heterogeneity (12%)		Tree health (8%)		76%
PdC	Humidity nutrient gradient (25%)	Dense, young forest (16%)	Conifer cover (15%)		Heterogeneous warm forest (12%)		Plant diversity (10%)		77%
PCA of landscape factors									
PsV	Habitat diversity (31%)	Modified areas (29%)	Open habitats (21%)		Distance to industry (19%)		–		84%
PdC	Habitat diversity (41%)	Distance to edges (28%)	–		–		–		70%

### Proportional β-diversity of moths

**Fig. 1:**
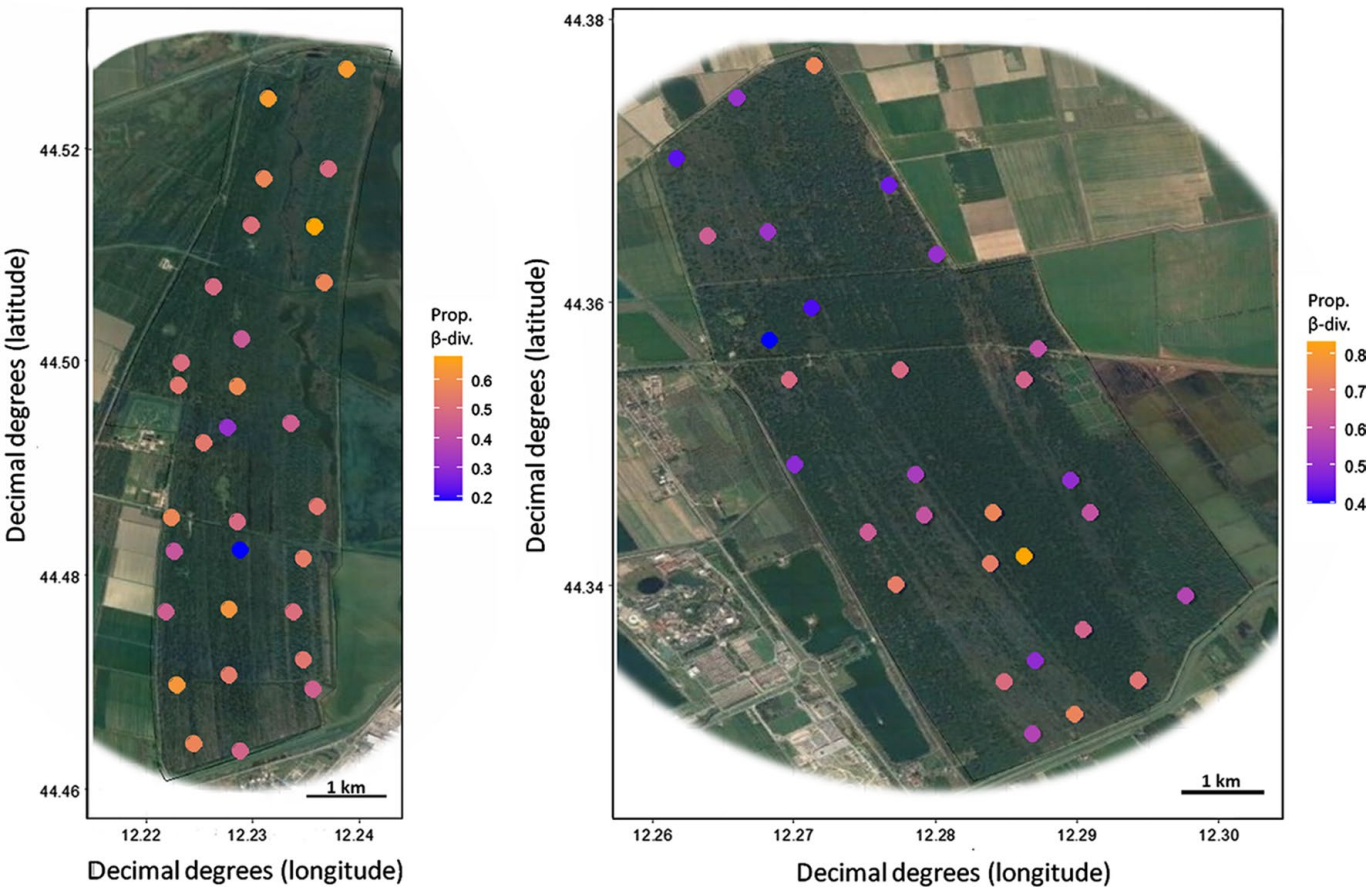
Distribution of the proportional β-diversity values of moth assemblages across the 60 sites in the two forest fragments, indicated by a color gradient. Orange = high proportional β-diversity, blue = low proportional β-diversity. Modified maps are based on Google™ satellite images. Left: Pineta san Vitale; right: Pineta di Classe

**Fig. 2:**
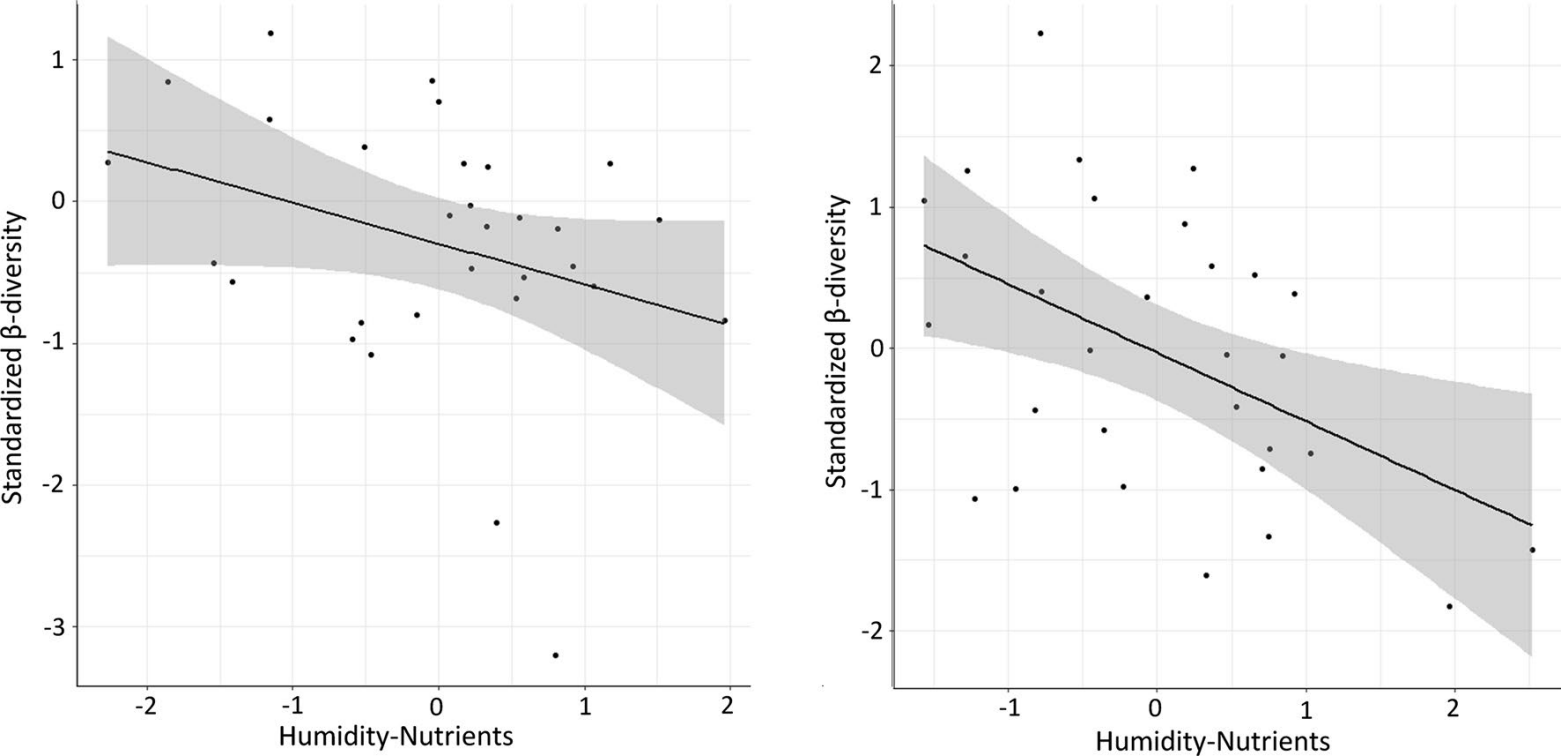
Bivariate correlations of proportional moth β-diversity with the two environmental factors which explained most of the variation in linear regression models. In the reserves Pineta san Vitale (left) and Pineta di Classe (right) the humidity-nutrient gradient was included in the best model. Grey shaded areas indicate the 95% confidence intervals of each model (black line)

In both reserves, there was strong variance in proportional moth β-diversity between individual sampling sites. In PsV proportional β-diversity ranged from 0.18 to 0.68 (mean: 0.52 ± 0.10). Smallest values were found at sites V14 and V20, located in the southern middle of the reserve, whereas we observed highest values in the north of the reserve, at sites V6, V1 and V2 (Fig. 1). For PdC, values ranged between 0.40 and 0.83 (Mean: 0.60 ± 0.11), showing in general higher proportional β-diversity, with smallest values at sites C7 and C8, and the highest value at C22 (Fig. 1). Overall, PsV had significantly lower proportional β-diversity compared to PdC (*t* = − 2.88, *p* = 0.01). So, inside PdC there was a substantially higher species turnover from site to site than in PsV. For assessing the potential influence of environmental variables on proportional β-diversity we first calculated two full linear models, separately for PsV and for PdC, including all predictors extracted through the respective PCAs. In these models, standardized β-diversity β_dev_ was used as the response variable. Through model selection via AIC, we then found the best models for PsV and PdC, respectively. In PsV, the PC-axis ‘Plant diversity’ (t = -2.37, beta-coefficient = -0.51, *p* = 0.03), the ‘Humidity-nutrient-gradient’ (*t* = − 2.33, beta-coefficient = -0.42, p = 0.03), the PC-axis ‘Modified areas’ (t = 2.10, beta-coefficient = 0.43, *p* = 0.05) and ‘Open habitats’ (*t* = − 1.53, beta-coefficient = − 0.29, *p* = 0.14) were included in the best model. Proportional β-diversity was lower at shady, nutrient-rich sites that provided high plant species richness (Fig. 2). Modified areas in the surroundings otherwise led to increased species turnover. This best model had an adjusted *R*^2^ value of 0.19. For PdC, the PC-axes ‘Humidity-nutrient-gradient’ (*t* = − 3.37, beta-coefficient = -0.48, p = 0.002), ‘Dense, young forest’ (*t* = 2.11, beta-coefficient = 0.30, *p* = 0.05), ‘Conifer cover’ (*t* = − 2.65, beta-coefficient = -0.38, *p* = 0.01) and ‘Heterogeneous warm forest’ (*t* = 1.34, beta-coefficient = 0.19, *p* = 0.19) were included in the best model. These four predictors altogether explained 42% of the among-site variation in proportional β-diversity of moth assemblages. Therefore, proportional β-diversity in PdC was lower at shady, humid and nutrient-rich sites (Fig. 2), but was also decreasing with an open, old-grown forest structure and more pine trees around. So, old grown conifer sites on humid and nutrient-rich ground had lower moth species turnover than younger, dry and dense forest sites.

### Differentiation diversity of moths

**Fig. 3:**
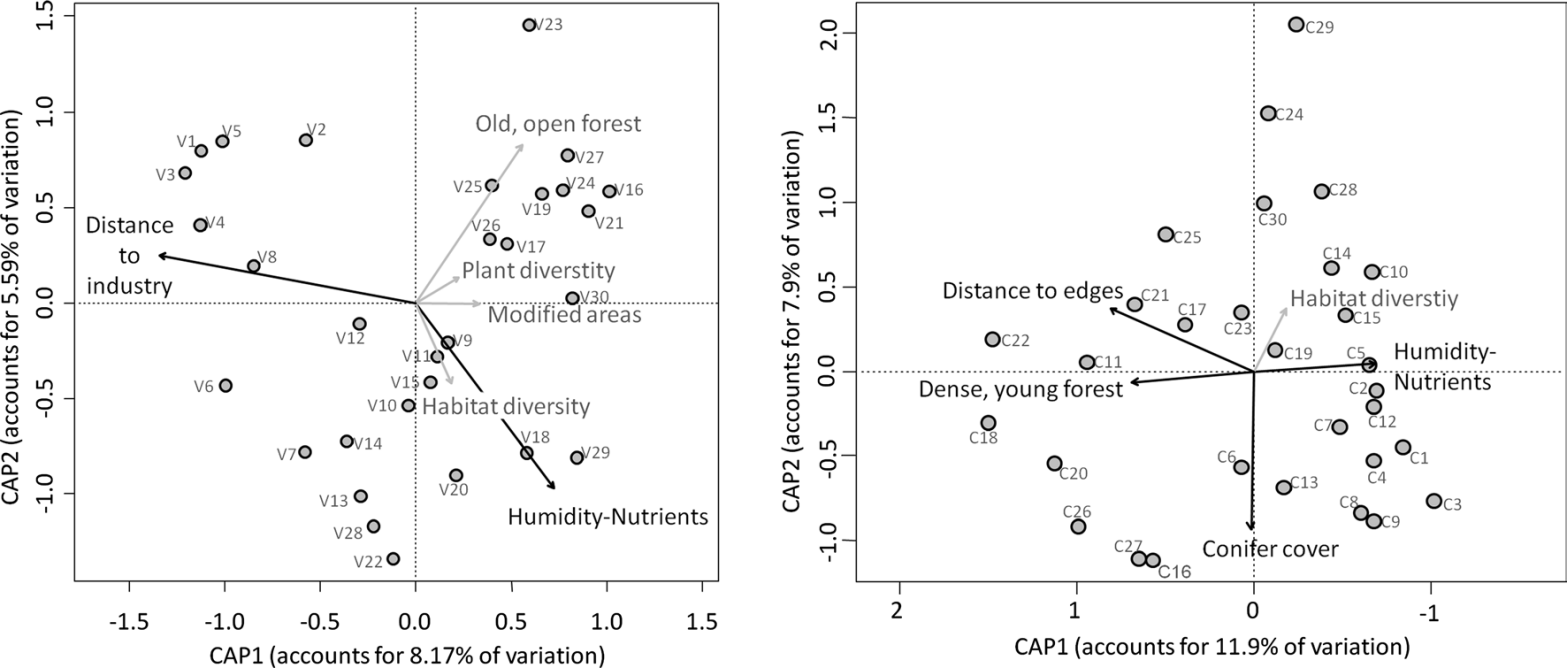
Canonical analysis of principal coordinates, separately for PsV (left) and PdC (right) moth communities. Environmental variables (the first three PC-axes of the local PCA, and selected PC-axes of the landscape PCA) are included as vectors. Significant predictors are colored in black, while those with minor effects on moth communities are colored grey. The results of the PERMANOVA, testing for how much of the variation in community composition is explained by the predictors and which of them had significant influences, are given in Table 3

Moth species composition differed significantly between PsV and PdC (PERMANOVA test: *R*^2^ = 0.12, *F*_1;58_ = 7.61, *p* = 0.001). This faunal distinction was mainly due to differences in relative species abundances between the two reserve fragments, while only a few species beyond the many singletons were exclusive to either PsV or PdC, respectively. In CAP analyses, local and landscape-scale variables explained more or less equal fractions of variation in moth community composition (Table 3). For PsV, 13.4% of the variation could be attributed to local factors, while 12.9% were explained by landscape factors. Here, we found the distance to the nearest industrial plant being a significant predictor of moth community composition, explaining about 5% of the total variation. The position of sites along the humidity-nutrient gradient also turned out to significantly shape moth species composition. Altogether, about 26.3% of the variation in moth community composition could be attributed to the investigated predictors (Table 3, Fig. 3).

**Table 3:** Results of PERMANOVA tests, checking for correlations between environmental variables and local moth community composition across 30 sites per reserve fragment

Reserve PsV				Reserve PdC			
	Factor	Explained variation (%)	*p* value		Factor	Explained variation (%)	*p* value
Local factors	Humidity-Nutrients	5.47	**0.01**	Local factors	Conifer cover	7.03	**0.001**
	Old, open forest	4.26	0.11		Dense, young forest	6.98	**0.004**
	Plant diversity	3.64	0.25		Humidity-nutrients	6.89	**0.003**
Landscape factors	Habitat diversity	3.49	0.31	Landscape factors	Habitat diversity	4.23	0.07
	Modified areas	4.10	0.14		Distance to edges	5.01	**0.02**
	Distance to industry	5.33	**0.02**				
Total		26.28		total		30.13	

The proportions of explained variation are given in brackets behind each axis

Percentages of explained variation by each environmental variable (PC-axes) in the CAP analyses are given as well as *p* values (based on 999 permutations)

Results printed in bold face were statistically significant (at *p* < 0.05) after table-wise False Discovery Rate correction

For PdC, the outcome of the constrained ordination analysis was remarkably different. 20.9% of the variation in the moth community could be explained by three local factors, all of which significantly shaping moth assemblages (Table 3). Additional 9.2% of the variation was attributable to two landscape-scale variables. Here, the ‘Distance to forest edges’ was a significant factor shaping moth community composition. In total, we were able to explain a slightly larger fraction (30.13%) of the variation in moth assemblage composition by the selected environmental descriptors in this second reserve fragment (Table 3, Fig. 3). Overall, the environmental factors that emerged as relevant correlates of local moth species composition varied strikingly between the two forest fractions.

## Discussion

The anciently connected forest areas of PsV and PdC nowadays show still similar basic environmental conditions. Most local site characters did not differ remarkably, although we found minor dissimilarity in plant diversity, forest structure and composition. Assuming that both forest patches basically are formed by the same environmental prerequisites (e.g. sandy underground, Mediterranean coastal climate), have a sufficiently large area and a quite compact shape, the preservation of the natural local habitats seems guaranteed (Petrášová-Šibíková et al. 2017). However, the current landscape context of both reserve fragments is very dissimilar. In PsV, there are more water canals than in PdC, indicating higher water availability. In fact, PsV is characterized by more humidity-indicating plants, while typical PdC plant species are affiliated with dry and warm conditions (Uhl et al. 2020a). Furthermore, sites in PsV are surrounded by much more near-natural areas—especially reed—resulting in higher landscape diversity, while the landscape context of sites situated in PdC is quite impoverished and simplified. This higher landscape diversity might be one reason for the higher moth species richness and α-diversity in PsV, compared to PdC (Uhl et al. 2020b), underlining the importance of landscape diversity for regional species richness in isolated conservation areas (Seibold et al. 2019).

### Proportional β-diversity

As a measure of diversity partitioning, proportional β-diversity can reveal the spatial scaling of diversity loss across sites (Socolar et al. 2016), however, studies of β-diversity are much less common than investigations on species richness or α-diversity (Mori et al. 2018). With increasing proportional β-diversity (i.e. ever-smaller species subsets), local communities become more heterogeneous, indicating subtractive heterogenization by the loss of ubiquitous species. Declining proportional β-diversity (larger species subsets), in contrast indicates community homogenization as rare species becoming more widespread (additive homogenization: Socolar et al. 2016). Analyzing β-diversity on a landscape-scale can furthermore reveal the processes of additive heterogenization, so increased turnover that is based on higher regional γ-diversity, and subtractive homogenization, meaning the disappearance of rare species on the landscape level (Socolar et al. 2016, Fig. 4). These latter two mechanisms describe the known co-variance of β-diversity and γ-diversity patterns, viz. the logical dependence of higher regional diversity favoring higher landscape-wide species turnover (Ulrich et al. 2017). Hence, additive heterogenization and subtractive homogenization cannot be found, when communities within one region are compared, as higher turnover mathematically is based on smaller species subsets. For insect communities, there are only a few β-diversity studies, mainly focusing on aquatic (Hepp et al. 2012; McCreadie and Adler 2018) and tropical insect communities (Beck et al. 2012; Kitching et al. 2013; Novotny et al. 2007) or conducted at much larger geographical scales (Chesters et al. 2019). Smaller-scale variation in β-diversity, especially in fragmented conservation areas, however, is until today only poorly understood.

**Fig. 4:**
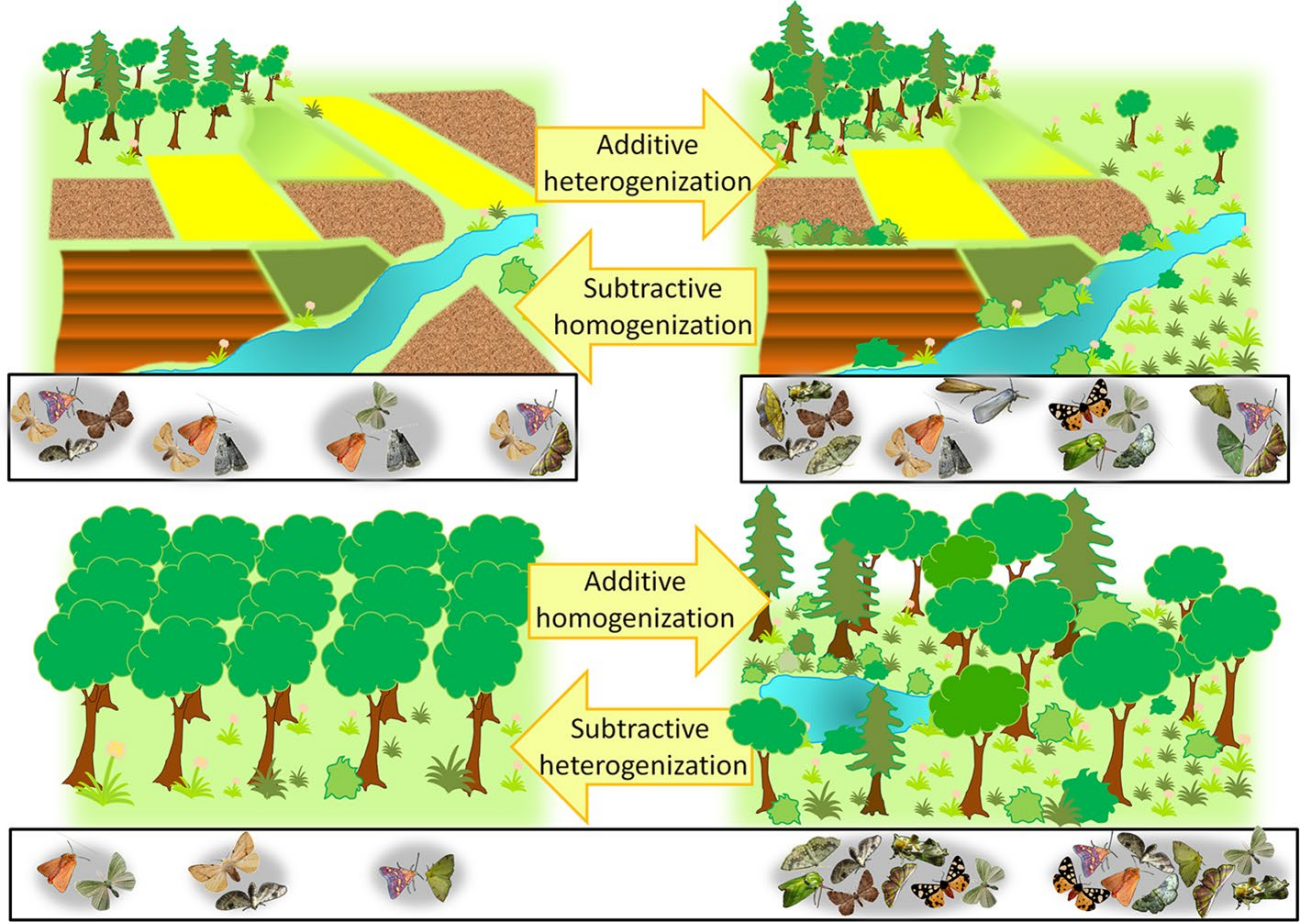
Schematic overview of the four processes that explain changes in β-diversity, following Socolar et al. (2016). Additive heterogenization and subtractive homogenization are acting on the landscape level and describe that with a declining regional species pool (represented by the white boxes, upper part), species turnover is also declining. So, in ever smaller species subsets (indicated by the grey background) the chance to observe the same species becomes higher. Additive homogenization and subtractive heterogenization, in contrast, are processes that are found when species subsets within one species pool (represented by only one white box, below) are compared. Within a forest, larger subsets of the species pool can be found where more niches are available, leading to lower proportional β-diversity

Comparing the two reserve fragments, moth assemblages in PsV were more homogenized with lower species turnover between sites. Since PsV comprises more different habitat types (Merloni and Piccoli 1999), one first might have expected the contrary. However, these diverse habitat structures and a well-developed forest understory can be found all over the reserve. In PdC, in contrast, some sites resemble PsV locations by their forest structure and their landscape context while other areas are structurally impoverished. At these locations a near-natural forest structure is still lacking, as well as any habitats other than mixed oak-pine stands in their vicinity. Since sites in PsV also harbor on average higher local moth species richness, we conclude that additive homogenization might have caused the lower species turnover in this northern reserve. Moth species that occurred rarely in PdC might be quite common in PsV, enhancing its mean α-diversity per site and reducing species turnover between sites.

Fitting to this assumption, proportional β-diversity in PdC was lower at shady, humid and nutrient-rich sites, negatively correlated to an increasing cover of conifer trees and to a more open, old-grown forest structure. So, larger diversity subsets in PdC occurred at well-developed forest sites, most resembling the ecological conditions of the PsV sites. In PsV, we observed the same correlation: Species turnover decreased along the ‘Humidity-nutrient gradient’, and with increasing plant diversity. Other local factors like the ‘Old, open forest’-axis were not included the best PsV model, although this factor was an equivalent for the ‘Dense, young forest’-axis in PdC. The PsV forest structure had overall less variance across sites compared to PdC. Very dense, young forest stands are missing here, and at most sites the amount of conifer and deciduous tree biomass is balanced. The gradient from younger, dense forest stands to old-grown open forest sites, therefore, is less pronounced than in PdC, what might be the reason for this factor being less relevant in PsV.

Forest succession is known to play a key role for insect communities. Looking at β-diversity, Miller and terHorst (2012) found that with ongoing succession there is a decreasing species turnover, supporting our own observations. Our study, however, furthermore points out, that additive homogenization seems to be key the process driving the decline in species turnover at near-natural forest sites. Formulated from another point of view, subtractive heterogenization, viz. the local lack of otherwise ubiquitous forest species at dense, young forest sites, may have caused the observed pattern (Fig. 4). So, additive homogenization and subtractive heterogenization here describe the same process, but in opposite directions (Fig. 4). However, this is only true for the interpretation of spatial analyses, as in time series, the direction of change from ancient to recent communities is fixed.

Landscape-scale aspects were not included in the best PdC-model. However, for PsV the ‘Open habitats’-axis and the ‘Modified areas’-axis seem to affect proportional β-diversity at least to some extent. The presence of open grasslands reduced species turnover, favoring additive homogenization through the establishment of specialized species in local communities. Near-natural habitat structures like open grasslands, therefore, can play a crucial role for insect β-diversity inside forests, as they break up the homogenous forest structure and provide more niches for different insects. In agricultural landscapes, Landis (2017) reviewed the important role of landscape complexity for maintaining high diversity and related ecosystem services. Our results, furthermore, corroborate the value of particular landscape structures for increasing species diversity inside conservation areas.

In contrast, we found higher species turnover between sites (smaller subsets) when an increasing amount of agricultural and urban areas was measured in the surroundings. This can indicate that (1) some species are missing at sites with more modified areas around (subtractive heterogenization), or—formulated again from another point of view (2) some species become more common when no anthropogenic land use in the surroundings can be found (additive homogenization) (Socolar et al. 2016). Human actions on the landscape scale can influence nature reserves indirectly, through the drift of pesticides (Zivan et al. 2016) or the influx of nutrients from surrounding agricultural landscapes. Nutrient input can alter vegetation structure (Uhl et al. 2020a) and also might reduce food plant quality for insects (Kurze et al. 2018). Additionally, light pollution can be enhanced when more urban areas are surrounding the sampling site. Artificial light at night has major effects on nocturnal insect communities, disrupts the development of insects at different life stages (Boyes et al. 2020), affects their fitness directly by reducing optical efficiency and orientation, and desynchronizes their internal clock (Owens and Lewis 2018). So, there are multiple possible reasons that might explain the observed patterns in β-diversity. Therefore, further studies are needed to more precisely unravel the effects of landscape-scale anthropogenic actions on nature reserves.

### Differentiation diversity

In contrast to proportional β-diversity, differentiation diversity is not a diversity partitioning metric but can be used to study the drivers of species composition (Jurasinski et al. 2009). Even though local site characteristics were rather similar between the two reserve fragments and there is a large basic moth species pool both reserves have in common, we found highly significant differences in moth species composition between PsV and PdC. Some species clearly are bound to the occurrence of particular food plants, e.g. *Eutelia adulatrix* exclusively occurs in PdC, because its larval food plant *Cotinus coggygria* can only be found there. As the two reserves have a quite diverged plant community in terms of species composition (Uhl et al. 2020a), this might explain some of the compositional differences in PsV and PdC moth communities. Other studies have established the influence of plant diversity on moth diversity (Root et al. 2017), and in fact also in the two study areas, a higher plant richness at the site scale level enhances local moth diversity (Uhl et al. 2020b). However, assuming that the particular composition of plant communities is often more important for local assemblages of herbivorous insects than the absolute number of plant species (Gavish et al. 2019; Kemp et al. 2017), this might be one reason for local predictors like the ‘Plant richness’-Axis failing to explain differences in moth community composition. From the landscape-level point of view, the presence of more reed areas in PsV might explain the occurrence of some specialist reed herbivores like *Phragmataecia castaneae* and *Schoenobius gigantella* as indicator species of PsV. The faunal differentiation between the two forest fragments is in line with the landscape-divergence hypothesis formulated by Laurance et al. (2007) who predicted that local communities tend to diverge when surrounded by different landscapes, even if the local conditions are not that different. Besides environment-driven deterministic processes, also ecological drift likely has contributed to differences in the moth communities (Gilbert and Levine 2017; Mori et al. 2018). As only a few of the recognized indicator species were exclusively found in one reserve, we conclude that for most species it was the difference in their abundances rendering them a statistical indicator for either PsV or PdC.

Small-scale insect community composition within the two reserve fragments was substantially influenced by local as well as landscape-scale factors, with roughly equal importance of both scales of effect. Accordingly, moths were again confirmed as suitable target organisms for small-scale analyses with distances of only about 500 m between sampling sites (Slade et al. 2013), although they are considered to be a quite mobile insect group. In contrast, other insect groups failed to reflect variation in vegetation structure and other environmental factors (Kemp et al. 2017). Emphasizing the importance of water and nutrient availability for Mediterranean plant and insect communities, only the humidity-nutrient gradient emerged as a significant predictor of moth species composition in both reserves. This PC-Axis well reflected the gradient from dry and warm sites to shady, humid and nutrient-rich forest locations with a rather dense canopy layer. However, ‘nutrient-rich’ does not mean that these sites were really eutrophic, as the highest average nutrient indicator values derived from the local vascular plant species lists in both reserves never exceeded a value of 5.51 (at location C1), indicating only moderate absolute nutrient availability. More likely, we interpret this PC-Axis as referring to a natural succession gradient. In a near-natural forest, shady sites with well developed sub-canopy layer, built up by small trees and shrubs, can regulate the local microclimate by buffering hot temperatures in summer as well as cold winter days (Prévosto et al. 2020). Furthermore, the forest humus layer ensures nutrient and water availability and again guarantees stable environmental conditions. These stable conditions, together with the structural richness of such near-natural forest sites positively affect moth taxonomic and functional diversity (Uhl 2020; Uhl et al. 2020b). That this also translates into an effect on community composition was therefore not surprising. Other forest structure components (like forest density and age, or conifer cover), however, only were significant predictors of variation in moth species composition in PdC. We again attribute this outcome to the reduced variance in PsV forest structure, where very dense, young forest stands and monotonous conifer sites were missing.

On the landscape-scale, especially the two anthropogenic influences emerged as significant predictors shaping moth communities inside the forest reserves. For sites in PsV, the distance to urbanized areas turned out to significantly affect differentiation β-diversity. Uhl et al. (2016) earlier demonstrated that the abundance of twelve ecologically informative micro-moth species was declining in the vicinity of the industrial plants in PsV, whereas only four species became more abundant there. In addition, in the present study some moth species were becoming significantly less abundant along with the ‘distance to industry’-Axis within PsV. For example, *Carpatolechia aenigma* (*r* = 0.57, *p* < 0.001) and also *Acrobasis consociella* (*r* = 0.42, *p* = 0.02) were less abundant in the vicinity to the industrial harbor. Larvae of these species feed on oak trees, which occur at all our study sites. The absence of these oak feeders in the south of PsV, therefore, might indicate locally poor food plant quality, as oaks near the industrial plants tend to have lower crown densities, indicating reduced fitness (Uhl and Wölfling 2015). Interestingly, some further specialized oak feeders were only observed in PdC. For example, *Catocala conversa* and *Spatalia argentina* never showed up in PsV during three summers of intense light-trapping efforts although local conditions seem favorable for both species. Furthermore, *Spatalia argentina* formerly occurred in PsV, as there are voucher specimens in old collections from around 1950 (Mirko Wölfling, unpublished observations). The current absence of these species might hint to some constraints acting on oak-feeding moth species in PsV. Our present study, therefore, confirms earlier findings of Uhl et al. (2016) on micromoths, but more concisely points out that the observed community shifts do not mainly refer to changes in local vegetation (Uhl et al. 2016), but seem to be influenced by landscape-scale drivers, indirectly affecting the food plant quality. The ‘Distance to industry’-gradient, however, may also be influenced by other landscape structures that were not analyzed. In the north of PsV, large reed areas exist. Though, they were not represented by any of the landscape factors, as they were too far away from the sampling sites. The proximity and amount of reed areas might have influenced the abundance of moths affiliated with *Phragmites australis* or aquatic plants, which were more likely to occur in the north of PsV. As an example, the aquatic species *Acentria ephemerella* (*r* = 0.40, *p* = 0.03) and also the reed affiliated species *Leucania obsoleta* (*r* = 0.49, *p* = 0.006) were significantly correlated to the ‘Distance to industry’-axis.

Looking at PdC, another anthropogenic landscape factor significantly affected moth communities. The distance to forest edges, which also represented a decreasing proportion of human-modified areas in the surroundings of the sampling sites, significantly shaped moth assemblages. The small differences in species composition here can be explained by possible spill-over of moths from surrounding ruderal or agricultural areas. Potential pest species like *Ostrinia nubilalis* (*r* = − 0.58, *p* < 0.001) and *Agrotis ipsilon* (*r* = − 0.60, *p* < 0.001) became significantly more abundant at PdC forest edges. Same was observed for *Dypterygia scabriuscula* (*r* = − 0.65, p < 0.001) and *Timandra comae* (*r* = − 0.59, *p* < 0.001), which both are feeding on *Rumex* species at ruderal sites as larvae. Conversely, forest species like *Macaria liturata* (*r* = 0.40, *p* = 0.03) or *Scoparia basistrigalis* (*r* = 0.50, *p* = 0.004), become less abundant at edge sided locations. Similar landscape-modulated edge effects on moth community composition were also described by Fuentes-Montemayor et al. (2012) who found especially woodland species to be dependent on larger forest fragments and forest centers. In small woodland patches and edges, forest species seem to be replaced by generalist species, confirming the species-replacement-hypothesis sensu Summerville and Crist (2003). So, anthropogenically induced modifications on landscape-scale, like land-use intensification or landscape simplification, do not only affect communities on site, but also more distant biota inside nature reserves. Our study shows that these landscape-scale effects are also detectable via small-scaled variation in community composition inside two forest nature reserve fragments. For the conservation of specialized forest species, it, therefore, seems especially important to preserve larger fragments of near-natural forest, with fewer edges between the reserve and modified areas. Structural heterogeneity within the forest, through the presence of other habitat structures like open grassland areas, furthermore can stabilize local communities and counteract biodiversity decline.

## Concluding remarks

Our results show that the variation of proportional β-diversity strongly depended on site-specific environmental gradients. The stronger these gradients are pronounced, the more likely they are to be reflected by proportional β-diversity and species turnover. The strength of gradients therefore always determines their importance for insect community composition and should always be considered when ecological data are interpreted.

Landscape attributes again emerged as important for the integrity of biota in forest fragments. Even in mobile insects such as moths, small-scaled community variation turned out to be related to both, local and landscape-scale environmental factors. In our study, a near-natural forest structure came up as the most important factor on the local scale, while on the landscape-scale, human modifications severely influenced community assembly of moths within nature reserves. Human actions therefore do not end at the field border and their effects on nearby protected natural habitats always need to be considered in conservation management.

## Supplementary Information

Below is the link to the electronic supplementary material.

Supplementary file1 (DOCX 14 KB)

Supplementary file2 (DOCX 13 KB)
