# Supplementary material for: From forest to fragment: compositional differences inside coastal forest moth assemblages and their environmental correlates
Source: Oecologia. 2021 Feb 1;195(2):453–67. doi: 10.1007/s00442-021-04861-7 (PMC7882585; doi:10.1007/s00442-021-04861-7)
Supplement: Supplementary file 1 — Supplementary file1 (DOCX 14 KB) [file 442_2021_4861_MOESM1_ESM.docx]

**Online Resource 2:** Overview over the factor loadings obtained in the three local factor PCAs. Values of <-0.5 or >0.5 are marked in bold. λ: eigenvalues of the respective PC-axes.

| Local factors  (PsV) | Old, open forest λ=2.84 | Plant species diversity λ=2.46 | Humidity-nutrient-gradient λ=2.45 | Herb-layer heterogeneity λ=1.69 | Tree health λ=1.16 |
| --- | --- | --- | --- | --- | --- |
| Plant species richness | 0.03 | 0.47 | 0.41 | 0.09 | -0.35 |
| Functional dispersion of plant species | -0.03 | **0.87** | -0.12 | -0.03 | 0.03 |
| Herb layer heterogeneity | 0.04 | -0.01 | 0.16 | **0.87** | -0.07 |
| Shrub layer heterogeneity | -0.45 | **0.61** | -0.14 | 0.27 | 0.00 |
| Ellenberg indicator “Humidity” | 0.14 | -0.29 | **0.89** | 0.05 | 0.05 |
| Ellenberg indicator “Nutrients” | 0.07 | -0.02 | **0.89** | -0.04 | 0.00 |
| Ellenberg indicator “Temperature” | **-0.53** | **0.50** | -0.42 | 0.04 | -0.20 |
| Forest density | **-0.84** | -0.20 | 0.02 | 0.28 | 0.16 |
| Canopy density | -0.16 | -0.06 | **0.66** | 0.45 | -0.01 |
| Cover of deciduous trees | -0.26 | **-0.76** | 0.12 | -0.02 | -0.10 |
| Cover of conifer trees | 0.42 | 0.37 | -0.08 | **0.66** | 0.18 |
| Mean basal area | **0.89** | -0.09 | 0.08 | 0.22 | 0.03 |
| Standard deviation of basal area | **0.75** | 0.04 | 0.00 | 0.28 | 0.22 |
| % dead standing trees | 0.08 | 0.06 | 0.04 | 0.01 | **0.93** |
| Local factors  (only PdC) | Humidity-nutrient-gradient λ=3.44 | Dense, young forest λ=2.27 | Conifer cover λ=2.03 | Heterogeneous, warm forest λ=1.63 | Plant diversity λ=1.39 |
| Plant species richness | 0.05 | -0.17 | 0.34 | -0.07 | **0.70** |
| Functional dispersion of plants | -0.31 | -0.09 | **0.75** | -0.22 | 0.13 |
| Herb layer heterogeneity | -0.45 | **0.66** | -0.03 | 0.26 | -0.02 |
| Shrub layer heterogeneity | -0.33 | 0.14 | -0.09 | -0.12 | **0.78** |
| Ellenberg indicator “Humidity” | **0.87** | -0.03 | -0.18 | 0.05 | -0.05 |
| Ellenberg indicator “Nutrients” | **0.88** | -0.01 | -0.11 | 0.03 | -0.08 |
| Ellenberg indicator “Temperature” | -0.46 | 0.32 | -0.18 | **0.58** | -0.19 |
| Forest density | 0.11 | **0.80** | 0.27 | 0.04 | -0.22 |
| Canopy density | **0.79** | 0.15 | 0.23 | -0.11 | -0.34 |
| Cover of deciduous trees | **0.70** | -0.04 | -0.30 | 0.30 | 0.01 |
| Cover of conifer trees | -0.08 | 0.14 | **0.88** | 0.26 | 0.04 |
| Mean basal area | 0.28 | **-0.64** | **0.50** | 0.38 | 0.16 |
| Standard deviation of basal area | 0.23 | -0.01 | 0.16 | **0.92** | -0.10 |
| % dead standing trees | -0.16 | **-0.76** | 0.08 | -0.02 | -0.19 |
