# Supplementary material for: From forest to fragment: compositional differences inside coastal forest moth assemblages and their environmental correlates
Source: Oecologia. 2021 Feb 1;195(2):453–67. doi: 10.1007/s00442-021-04861-7 (PMC7882585; doi:10.1007/s00442-021-04861-7)
Supplement: Supplementary file 2 — Supplementary file2 (DOCX 13 KB) [file 442_2021_4861_MOESM2_ESM.docx]

**Online Resource 3:** Overview over the factor loadings of the three landscape scale PCAs. Values of <-0.5 or >0.5 are marked in bold. λ: eigenvalues of the respective PC-axes.

| Landscape factors  (only for PsV) | Habitat diversity  λ=2.07 | Modified areas  λ=1.95 | Open habitats  λ=1.42 | Distance to industry  λ=1.25 |
| --- | --- | --- | --- | --- |
| Distance to reserve edge | **-0.48** | -0.34 | 0.28 | 0.40 |
| Distance to canal | -0.19 | **-0.60** | 0.30 | 0.32 |
| Distance to industry | -0.02 | 0.01 | -0.07 | **0.96** |
| Diversity of natural habitats (200m) | **0.92** | 0.14 | 0.32 | -0.07 |
| Edge density (500m) | 0.39 | **0.79** | -0.21 | 0.25 |
| Proportion of reed (200m) | **0.90** | 0.04 | -0.36 | 0.02 |
| Proportion of grassland (200m) | -0.03 | 0.01 | **0.98** | -0.04 |
| Proportion of modified areas (500m) | -0.07 | **0.91** | -0.02 | -0.06 |
| Landscape factors  (only for PdC) | Habitat diversity  λ=3.31 | Distance to edges  λ=2.27 |  |  |
| Distance to reserve edge | -0.26 | **0.85** |  |  |
| Distance to canal | **-0.53** | 0.40 |  |  |
| Distance to industry | 0.23 | **0.72** |  |  |
| Diversity of natural habitats (200m) | **0.93** | -0.15 |  |  |
| Edge density (500m) | **0.78** | -0.26 |  |  |
| Proportion of reed (200m) | **0.86** | -0.06 |  |  |
| Proportion of grassland (200m) | **0.75** | 0.06 |  |  |
| Proportion of modified areas (500m) | 0.36 | **-0.88** |  |  |
